# The Editorial of the Special Issue “Diagnosis of Lower Genital Tract Disease”

**DOI:** 10.3390/diagnostics13152515

**Published:** 2023-07-28

**Authors:** Fabio Bottari, Anna Daniela Iacobone

**Affiliations:** 1 Division of Laboratory Medicine, Istituto Europeo di Oncologia IRCCS, 20145 Milan, Italy; 2 Preventive Gynecology Unit, Istituto Europeo di Oncologia IRCCS, 20145 Milan, Italy; annadaniela.iacobone@ieo.it

A range of conditions involving the vulvovaginal and anal area, and those associated with human papillomavirus (HPV) infection, which can manifest as benign, pre-neoplastic, or neoplastic lesions, can be grouped into lower genital tract diseases. Currently, there is growing interest in innovative approaches for diagnosing and preventing cervical cancer through primary human papillomavirus (HPV) screening programs. However, it is important to note that other lower genital tract diseases are not related to HPV infection. Consequently, cytology, colposcopy, and sonography might play a crucial role in their assessment and management.

The objective of this Special Issue is to address current and emerging issues in the field of the diagnosis, assessment, staging, and management of both HPV-related and unrelated pathologies of the lower ano-genital tract. Topics of interest include advancements in HPV testing and laboratory biomarkers, investigations into the actual utility of cytology, the implementation of diagnostic tools to facilitate differential diagnosis, and the current clinical management of lower genital tract diseases.

This Special Issue has had the pleasure of receiving articles that cover all the requested fields of lower ano-genital tract disease. In total, ten original articles and one systematic review have been presented.

The first article submitted to the Special Issue demonstrates how a diagnostic tool commonly used in the field of ophthalmology can be adapted to effectively evaluate vaginal dryness. Gabrieli et al. [1] presented their findings on the “modified Schirmer test,” which serves as an objective scale for assessing vaginal moisture levels. This study highlights the test’s potential to manage women who experience vaginal dryness.

Furthermore, in line with the theme of the Special Issue, several articles focused on HPV tests have been included. These articles explore diagnostic innovations such as self-sampling [2] and mRNA-based HPV tests [3]. Additionally, there is an article investigating the usefulness of HPV testing as a test-of-cure in post-treatment follow-up [4].

Martinelli et al. published an article presenting promising results from their pilot study on the use of the Onclarity HPV assay on vaginal samples and first-void urine samples. The accuracy of HPV testing on self-collected samples was found to be nearly equivalent to clinician-collected cervical samples. This finding represents the basis on which several studies have been conducted to validate HPV tests on self-sampling as a diagnostic tool to expand cervical cancer screening coverage [2].

Nikolic et al. showed that mRNA testing may be more relevant than HPV DNA testing for the assessment of lesion grade and in the diagnosis and monitoring of women at risk of progressive cervical disease. The HPV mRNA test shows great potential as both a screening test and a triage test for HPV DNA-positive women due to its higher specificity (detecting active infections) and greater objectivity compared to cytology. Employing the mRNA test for the improved risk stratification of HPV infection could help reduce unnecessary examinations, lower costs, and alleviate patient anxiety [3].

Bottari et al. provided evidence that the HPV test serves as a valid option for test-of-cure monitoring in patients treated for CIN2+ lesions during follow-up. Their article also emphasizes the importance of using validated HPV DNA or RNA tests, as they consistently produce comparable results. Non-validated tests do not share the same assurance, emphasizing the strong recommendation to utilize validated HPV tests exclusively in both screening and test-of-cure settings [4].

Other intriguing articles in this Special Issue focus on the use of diagnostic imaging for the diagnosis of genital diseases, particularly in the early stages of cervical cancer [5] and endometrial carcinoma [6].

Vidal Urbinati et al. demonstrated a significant correlation between vaginosonography and magnetic resonance imaging in the assessment of tumor dimensions. They highlighted the superior performance of the sonographic tool in detecting small tumors and predicting the absence of fornix infiltration [5].

In their retrospective study, Pace et al. identified several ultrasonographic features that, in conjunction with subjective hysteroscopic assessment by experienced clinicians, can suggest the presence of occult endometrial cancer in patients with a preoperative histologic diagnosis of atypical endometrial hyperplasia. These features include endometrial thickness, a larger diameter of the lesion, an interrupted endometrial–myometrial junction, and high vascular density on color Doppler imaging [6].

Furthermore, a significant section of this Special Issue is devoted to the pathology of the lower extra-cervical genital tract. Within this context, three original articles and one review have been published, focusing on various aspects such as vaginal pre-cancers and cancer [7], extramammary Paget’s disease of the vulva [8], anal cancer [9], and bladder cancer [10].

The retrospective analysis conducted by Iacobone et al. aimed to investigate the correlation between colposcopic features and the development of high-grade vaginal intraepithelial neoplasia (VAIN) [7]. An accurate diagnosis is crucial for the effective management of vaginal pre-cancers and cancers. The study found that certain colposcopic findings, such as grade 2, papillary, and vascular patterns, can serve as predictive factors for high-grade VAIN. Moreover, HPV genotyping can contribute to risk stratification and facilitate the prompt identification of women at a higher risk of persistence, recurrence, and progression to vaginal cancer in cases of high-grade VAIN.

Another highly intriguing article authored by Iacobone et al. [8] explores cervico-vaginal involvement in extramammary Paget’s disease of the vulva, a distinctive and uncommon condition. This article provides a comprehensive examination of both the diagnostic aspects and the management strategies related to this pathology, highlighting the importance of recognizing its significance in women with a long-standing history of vulvar Paget’s disease. The article emphasizes the value and reliability of cytology, coupled with immunocytochemistry, as a prompt and indispensable tool for early diagnosis.

In this Special Issue, Guarendian et al. [9] employed whole-genome sequencing, an innovative method that is transforming tumor genetics management. Their findings, utilizing this advanced approach, indicate that HPV 16 sub-lineages do not exhibit association with disease versus asymptomatic carriage, nor do they demonstrate independent associations with outcomes in anal cancer patients.

Additionally, a captivating systematic review on the prevalence of HPV infection in bladder cancer is presented within this Special Issue. In this article, Muresu et al. highlight a potential role of HPV in the development of bladder cancer, providing indirect support for the implementation of primary preventive strategies as recommended by international authorities and study groups [10].

Finally, the article by Origoni et al. draws attention to the fundamental role of education in medicine, particularly in the training of colposcopists. This study emphasizes the significance of educating new generations of skilled gynecologists and standardizing a procedure that is inherently subjective. The adoption of colposcopy standards and quality recommendations by scientific societies is a fundamental step towards ensuring effective cervical cancer prevention [11].

All the papers presented in this Special Issue underscore the significance of research and progress in the diagnosis and management of lower genital tract diseases. The prevailing challenge lies in the exploration of increasingly sophisticated and precise tools that allow for early detection and robust management approaches.

The advancement of diagnostic techniques and the evolution of patient management hold promising potential to alleviate the burden and enhance the treatment of lower genital tract diseases.
